# New species of the *Pseudancistrus barbatus* group (Siluriformes, Loricariidae) with comments on its biogeography and dispersal routes

**DOI:** 10.3897/zookeys.406.7011

**Published:** 2014-04-29

**Authors:** Gabriel de Souza da Costa e Silva, Fábio Fernandes Roxo, Ricardo Britzke, Claudio Oliveira

**Affiliations:** 1 Laboratório de Biologia e Genética de Peixes, Departamento de Morfologia, IB-UNESP, Campus de Botucatu, 18618-000, Botucatu, SP, Brazil

**Keywords:** Ancistrini, freshwater, molecular phylogeny, F-reticulon 4, Brazilian Shield

## Abstract

A new species of *Pseudancistrus* is described from the Tapajós Basin, and assigned to the *P. barbatus* group by having hypertrophied odontodes along the snout and lacking evertible cheek plates. The new species is distinguished from other species in that group (*P. barbatus*, *P. corantijniensis*, *P. depressus* and *P. nigrescens*) by its pattern of spots, length and color of snout odontodes, greater head depth, cleithral width, anal-fin spine length, peduncle depth and internares width. Molecular phylogenetic results corroborate placement of the new species in the *Pseudancistrus barbatus* group which is otherwise distributed in the Xingu Basin and rivers draining the Guyana Shield into the Atlantic Ocean. Topology tests strongly reject alternative hypotheses supporting close relationships with *Guyanancistrus*, *Lithoxancistrus* or the species *Pseudancistrus pectegenitor*, *P. sidereus* and *P. genisetiger*. Additionally, we propose two hypotheses on the distribution of the new species in the rio Tapajós, a Brazilian Shield drainage. The first one proposes that ancestral stock of the *P. barbatus* group was widely distributed throughout rivers draining the Guyana and Brazilian shields, and the species *P. zawadzkii* and *Pseudancistrus* sp. L17 are in the limit of the distribution for the group in Tapajós and Xingu rivers. The second hypothesis proposes that ancestral stock of the *P. barbatus* group was restricted to Guyana Shield rivers, and that headwater capture events permitted several dispersal routs through Guyana and Amazon rivers, permitted that the ancestral lineages of *Pseudancistrus* sp. L17 and *P. zawadzkii* reached the rivers of Amazon basin.

## Introduction

Ancistrini is a highly diverse tribe of the subfamily Hypostominae, with 30 genera (Lujan and Armbruster 2011; Covain and Fisch-Muller 2012; Salcedo 2013) and 252 valid species (Eschmeyer and Fong 2013) widely distributed in the Neotropics from rivers in Panamá to the La Plata system in Argentina. Armbruster (2004a) provided morphological support for the monophyly of Ancistrini based on his extensive analysis of relationships within Loricariidae. Molecular data, however, suggested that Ancistrini is not monophyletic (Montoya-Burgos 1998; Covain and Fish-Muller 2012).

Species of the genus *Pseudancistrus* Bleeker, 1862 are distributed in the Orinoco, Amazon and Jaguaribe river systems, and rivers draining the Guyana Shield into the Atlantic Ocean. Armbruster (2004a) recognized *Pseudancistrus* as a monophyletic group and included *Guyanancistrus* Isbrücker, Seidel, Michels, Schraml & Werner, 2001 and *Lithoxancistrus* Isbrücker, Nijssen & Cala, 1988 in its synonymy. Based on molecular and morphological data, Chambrier and Montoya-Burgos (2008) defined a subgroup within *Pseudancistrus* called the *Pseudancistrus barbatus* group and composed of *Pseudancistrus barbatus* (Valenciennes, 1840), *Pseudancistrus depressus* (Günther, 1868), *Pseudancistrus nigrescens* Eigenmann, 1912, and *Pseudancistrus corantijniensis* De Chambrier & Montoya-Burgos, 2008. That group was morphologically defined by having hypertrophied odontodes along the snout and lacking evertible cheek plates. Recently, Covain and Fisch-Muller (2012) suggested that *Pseudancistrus guentheri* (Regan, 1904) and *Pseudancistrus kwinti* Willink, Mol & Chernoff, 2010 may be added to the *Pseudancistrus barbatus* group. Covain and Fisch-Muller (2012) also recognized *Pseudancistrus* as paraphyletic, and restricted the genus by the *Pseudancistrus barbatus* group. They revalidated the genera *Guyanancistrus* and *Lithoxancistrus*, and considered *Pseudancistrus pectegenitor* Lujan, Armbruster & Sabaj Pérez, 2007, *Pseudancistrus sidereus* Armbruster, 2004b, and *Pseudancistrus genisetiger* Fowler, 1941 to represent two separate lineages unrelated to *Pseudancistrus*. Covain and Fisch-Muller (2012) suggested that these two lineages represent undescribed genera.

In this paper, we present a formal description of a new species of *Pseudancistrus* from the Tapajós river basin. Additionally, we provide a phylogenetic context for the new species based on analysis of sequence data of F-reticulon 4 nuclear gene, and a brief discussion of biogeographic scenarios that may explain the distribution of the new species in the rio Tapajós and northern Brazilian Shield.

## Material and methods

### Sampling and morphological analysis

After capture, fish were anesthetized using 1% benzocaine in water, and either preserved in 95% ethanol for molecular studies or fixed in 10% formaldehyde for morphological studies. Vouchers and tissues were deposited in the collection of the Laboratório de Biologia e Genética de Peixes (LBP) and Museu de Zoologia da Universidade de São Paulo (MZUSP), Brazil, Muséum d’histoire naturelle de la ville de Genève (MHNG), Switzerland, Academy of Natural Sciences of Philadelphia (ANSP) and Auburn University (AUM), U.S.A., and Smithsonian Tropical Research Institute (STRI), Panama. Measurements and counts were taken on left side of specimens. Measurements follow Armbruster (2003), and were taken point to point to the nearest 0.1 mm with digital calipers.

### DNA sequencing

Total DNA was extracted from ethanol-preserved muscle, fin, and liver samples using the Wizard Genomic DNA Purification Kit (Promega, Madison, Wisconsin, U.S.A.). Partial sequences of F-reticulon 4 were amplified using polymerase chain reaction (PCR) with the following primers from Chiachio et al. (2008): Freticul4-D 5’-AGG CTA ACT CGC TYT SGG CTT TG-3’, Freticul4-R 5’-GGC AVA GRG CRA ART CCA TCT C-3’, Freticul4 D2 5’-CTT TGG TTC GGA ATG GAA AC-3’, Freticul4 R2 5’-AAR TCC ATC TCA CGC AGG A-3’, Freticul4 iR 5’-AGG CTC TGC AGT TTC TCT AG-3’.

Amplifications were performed in a total volume of 12.5 μl containing 1.25 μl of 10X PCR buffer (20 mM Tris-HCl, pH 8.0, 40 mM NaCl, 2 mM Sodium Phosphate, 0.1 mM EDTA, 1 mM DTT, stabilizers, 50% (v/v) glycerol), 0.375 μl MgCl2 (50nM), 0.25 μl dNTPs (2 nM), 0.25 μl (each 5 mM primer), 0.05 μl Platinum® Taq DNA Polymerase (Invitrogen), 1 μl template DNA (50 ng), and 9.075 μl ddH2O. The nuclear markers were amplified in two PCR experiments; the first amplification using the primers Freticul4-D and Freticul4-R for 37–40 cycles (30 sec at 95°C, 30 sec at 48°C, and 135 sec at 72°C); and the second amplification using the primers Freticul4 D2, Freticul4 R2, and Freticul4 iR for 37–40 cycles (30 sec at 95°C, 30 sec at 53–54°C, and 135 sec at 72°C).

The products were then identified on a 1% agarose gel. The PCR products were purified using ExoSap-IT® (USB, Affymetrix Corporation, Cleveland, Ohio) following the manufacturer’s instructions. The purified PCR products were used to make a sequencing PCR using the BigDyeTM Terminator v 3.1 Cycle Sequencing Ready Reaction Kit (Applied Biosystems- Life Technologies do Brasil Ltda, Vila Guarani, SP, Brazil). Subsequently, the amplified DNA was purified again and loaded onto a 3130-Genetic Analyzer automatic sequencer (Applied Biosystems), in the Instituto de Biociências, Universidade Estadual Paulista, Botucatu, São Paulo. Contigs were assembled and edited in BioEdit 7.0.9.0 (Hall 1999). Where uncertainty of nucleotide identity was detected, IUPAC ambiguity codes were applied. All sequences obtained in this study were deposited in GenBank (Table 3).

### Sequence alignment and phylogenetic analyses

The DNA sequences were aligned using ClustalW program implemented in DAMBE 5.2.31 (Xia and Xie 2001) and edited in BioEdit 7.0.1 (Hall 1999), using default parameters. The alignments were inspected by eye for any obvious misalignments that were then corrected. Alignment errors only were changed where indels of 1 bp were added to introns of the reticulon gene. The sequence of F-reticulon 4 of the new species was sequenced twice, and a preliminary phylogenetic analysis was performed to control potential sequencing errors involving pseudogenes, paralogous copies or laboratory cross-contamination or mistakes during manipulations of samples. Nucleotide variation was examined using MEGA 5.0 (Tamura et al. 2007). To evaluate the occurrence of substitution saturation, we estimated the index of substitution saturation (Iss) in DAMBE 5.2.31 (Xia and Xie 2001), as described by Xia et al. (2003) and Xia and Lemey (2009).

Maximum-Likelihood (ML) analyses were performed using RAxML Web-Servers (Randomized Accelerated Maximum Likelihood, Stamatakis et al. 2008) which implements a faster algorithm of heuristic search with bootstrap pseudoreplicates (RBS). Bootstrap resampling (Felsenstein 1985) was applied to assess support for individual nodes using 1,000 replicates. Random starting trees were used for each independent ML tree search and all other parameters were set on default values. The ML analysis was conducted under a Generalized Time Reversible (GTR) model, with Gamma distribution (G) and Invariable Sites according to Modeltest 3.7 results (Posada and Crandall 1998). Gaps were treated as missing data.

Alternative tree topologies were evaluated in the program Treefinder (Jobb et al. 2004) using the Shimodaira and Hasegawa (SH) test (Shimodaira and Hasegawa 1999), the Approximately Unbiased (AU) test (Shimodaira 2002), and the Expected Likelihood Weights (ELW) method (Strimmer and Rambaut 2002). All tests were conducted under ML with a GTR model and Gamma distribution.

## Results

### Pseudancistrus zawadzkii sp. n.

http://zoobank.org/F244A7A4-253A-49B8-B027-16B640FDBCCF

http://species-id.net/wiki/Pseudancistrus_zawadzkii

Figure 1 , Table 1

#### Holotype.

MZUSP 115056, male, 116.4 mm SL. Brazil: Pará State: municipality of Itaituba: rio Tapajós (Amazon basin), 04°33'09.7"S, 56°17'59.6"W, 11 June 2012, R. Britzke and CEPTA’s team.

#### Paratypes.

Brazil: Pará State: municipality of Itaituba: LBP 15045 (2 females, 97.9−128.7 mm SL), LBP 17724 (1 female, 87.5 mm SL), collected with holotype; LBP 16195 (1 male, 116.4 mm SL), rio Tracuá (trib. rio Tapajós), 04°28'11.2"S, 56°17'01.1"W.

#### Diagnosis.

**Figure 1.**
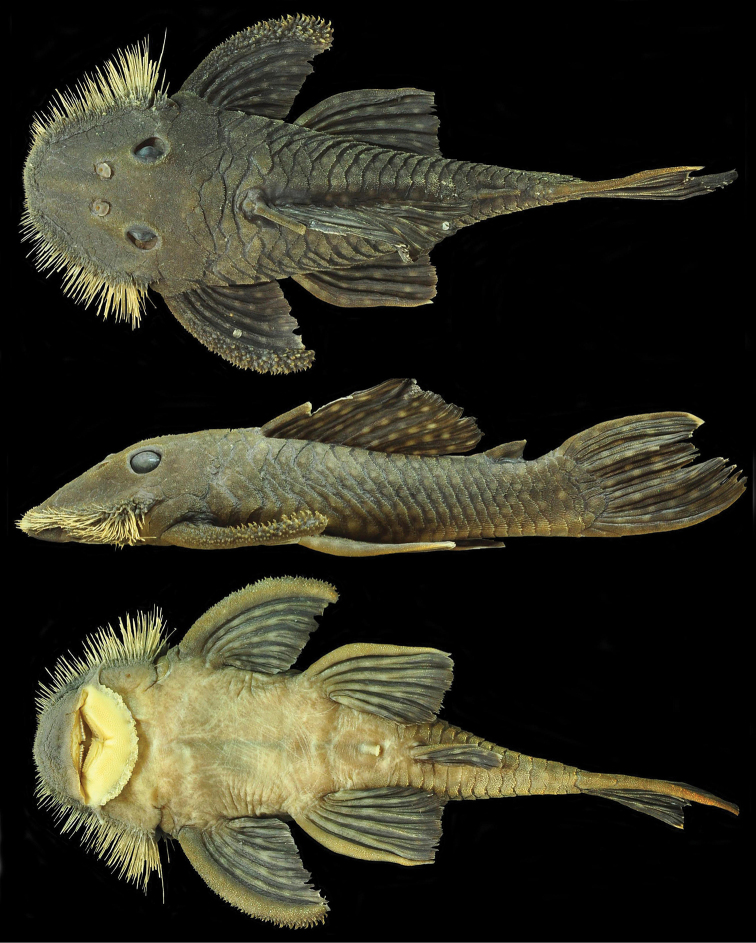
*Pseudancistrus zawadzkii*, MZUSP 115056, holotype, male, 116.4 mm SL; Pará State, Tapajós river basin, Brazil.

*Pseudancistrus zawadzkii* is distinguished from all congeners, except species of the *Pseudancistrus barbatus* group, by presence of hypertrophied odontodes along the snout margin and the lack of evertible cheek plates. It further differs from two members of that group, *Pseudancistrus barbatus* and *Pseudancistrus depressus*, by having whitish spots that abruptly increase in size between the head (diameter 1.1−1.3 mm) and body (diameter 2.6−3.0 mm) (vs. whitish spots very small on whole body less than 1 mm), and snout odontodes yellowish (vs. snout odontodes reddish-brown). The new species differs from the other two members of the *Pseudancistrus barbatus* group, *Pseudancistrus corantijniensis* and *Pseudancistrus nigrescens*, by having odontodes along margin of snout increasing gradually in length from posterior of snout tip to cheek (vs. length of snout odontodes more uniform, smaller on tip of snout) and by having odontodes relatively longer on the most posterior portion of the nonevertible check plates (Fig. 1) (vs. odontodes shorter) (see fig. 3 in Chambrier and Montoya-Burgos 2008 for comparison of both characters). Additionally, *Pseudancistrus zawadzkii* differs from *Pseudancistrus nigrescens* by having rounded spots that do not cover more than one plate along the body (vs. whitish spots that become hazier along the body and can cover more than one plate, see *Pseudancistrus nigrescens* in fig. 3 in Chambrier and Montoya-Burgos (2008). Moreover, *Pseudancistrus zawadzkii* is distinguished by having a greater head depth, 67.0−72.7% of HL (vs. 38.3−44.9% of HL in *Pseudancistrus barbatus*; 40.6−53.0% of HL in *Pseudancistrus corantijniensis*, data based on original description; and 52.5−56.6% of HL in *Pseudancistrus nigrescens*); greater cleithral width, 35.2−38.0% of SL (vs. 31.1−32.7% of SL in *Pseudancistrus nigrescens* and 29.7−33.4% of SL in *Pseudancistrus barbatus*); shorter distance between posteromedial margin of supraoccipital and origin of dorsal-fin, 6.7−9.2% of SL (vs. 10.4−11.6% of SL in *Pseudancistrus nigrescens*); greater anal-fin spine length, 11.9−13.8% of SL (vs. 7.3−10.4 of SL in *Pseudancistrus barbatus*); greater peduncle depth, 12.5−14.2% of SL (vs. 9.3−10.4 of SL in *Pseudancistrus barbatus*); and wider internares distance, 12.7−16.6% of HL (vs. 9.9−11.8% of HL in *Pseudancistrus barbatus*). *Pseudancistrus zawadzkii* differs from *Pseudancistrus kwinti* and *Pseudancistrus guentheri*, two probable members of *Pseudancistrus barbatus* group by having whitish spots of the body (vs. body mottled or with bars, in *Pseudancistrus kwinti* and body plates dark at the base and pale along the edges, in *Pseudancistrus guentheri*).

**Figure 2.**
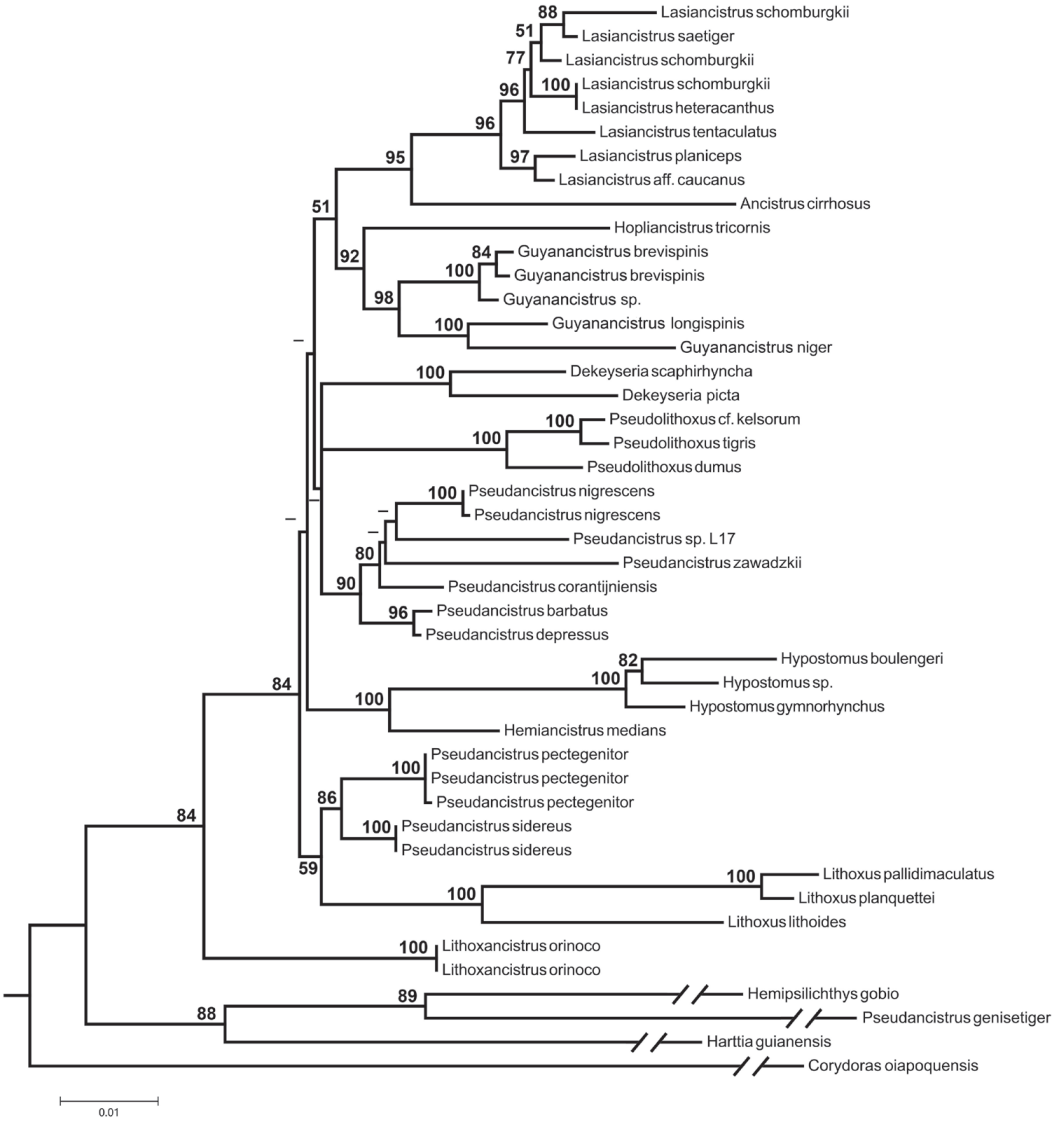
Maximum-likelihood tree based on nuclear gene sequence F-reticulon 4 (-lnL = 11470.59). Numbers next to nodes are bootstrap values based on 1,000 pseudoreplicates. Values below 50% are not shown.

#### Description.

Morphometric data presented in Table 1. In lateral view, dorsal profile convex from snout tip to dorsal-fin origin; straight, gradually descending from dorsal-fin origin to posterior insertion of adipose fin; straight, steeply ascending to insertion of caudal fin; ventral profile flat from snout tip to anal-fin origin; shallowly concave from anal-fin insertion to lower caudal-fin spine; greatest body depth at dorsal-fin origin. In dorsal view, greatest body width across cleithral region; snout broadly elliptical; body progressively narrowed from opercular region to caudal fin. Cross-section of body between pectoral and pelvic fins rounded dorsally and flattened ventrally; cross-section of caudal peduncle ellipsoid.

**Table 1.** Morphometric data for *Pseudancistrus zawadzkii*.

	*Pseudancistrus zawadzkii* n = 5			
	Holotype	Range	Mean	SD
**Standard length (SL)**	116.4	128.7−87.5	109.5	
**Percents of SL**				
Predorsal length	43.3	43.1−46.1	44.5	1.3
Head length	36.6	32.9−37.8	36.3	1.9
Head-dorsal length	6.7	6.7−9.2	8.1	1.2
Cleithral width	35.2	35.2−38.0	36.7	1.2
Head pectoral length	30.5	29.6−32.2	30.9	0.9
Thorax length	23.5	21.2−23.5	22.5	1.1
Pectoral-spine length	31.5	31.3−33.2	31.9	0.7
Abdominal length	24.2	22.6−26.1	24.3	1.3
Pelvic-spine length	28.4	25.6−28.4	27.2	1.2
Post-anal length	31.2	29.6−31.2	30.5	0.7
Anal-fin spine length	12.5	11.9−13.8	12.6	0.7
Dorsal pectoral depth	27.3	26.6−30.7	28.6	1.7
Dorsal spine length	24.7	24.7−29.9	27.5	2.3
Dorsal pelvic depth	22.9	22.1−26.4	24.1	1.7
Dorsal-fin base length	31.2	29.1−31.2	30.0	1.0
Dorsal-adipose distance	11.2	10.5−13.7	11.6	1.2
Adipose-spine length	7.8	6.79−8.78	7.8	0.7
Dorsal adipose caudal distance	11.7	11.7−15.6	13.7	1.7
Caudal peduncle depth	12.5	12.5−14.2	13.3	0.6
Ventral adipose caudal distance	22.9	22.9−25.3	23.9	1.0
Adipose anal distance	21.3	18.5−21.3	19.8	1.0
Dorsal-anal distance	16.0	15.8−17.8	16.8	0.8
Pelvic-dorsal distance	29.5	22.0−29.5	22.5	2.7
**Percents of head length (HL)**				
Head-eye length	29.4	28.1−30.1	29.1	0.8
Orbital diameter	14.6	14.5−18.8	15.8	1.7
Snout length	63.2	63.2−70.5	66.8	3.1
Internares width	14.4	12.7−16.6	14.4	1.4
Minimal interorbital distance	28.8	28.8−35.7	32.2	2.5
Mouth length	53.8	52.0−60.6	55.7	3.5
Barbel length	14.0	7.6−14.0	10.6	2.6
Dentary tooth cup length	17.6	17.0−19.6	18.5	1.1
Premaxillary tooth cup length	17.8	17.2−19.2	18.2	0.7
Head depth	68.9	67.0−72.7	68.8	2.3

Body almost entirely covered by plates; ventral portions of head and abdomen and dorsal-fin base naked. Five lateral rows of dermal plates, dorsal plates 21−24, lateral mid-dorsal plates 19−21, lateral median plates 22−24, lateral mid-ventral plates 21−24, lateral ventral plates 18−20. Three predorsal plates; eight plates below dorsal-fin base; four plates between dorsal fin and adipose fin; five rows of plates on caudal peduncle. Dorsal spinelet present.

Body plates and cleithrum have minute odontodes. Odontodes slightly hypertrophied on pectoral-fin spines, becoming gradually larger towards tips. Numerous yellowish hypertrophied odontodes along lateral margins of head including snout; odontodes small on tip of snout, increasing gradually in length from anterolateral margin of snout to cheeks; longest odontodes on posterior most portion of non-evertible cheek plates. Eyes small (orbital diameter 14.5−18.8% of HL), dorsolaterally positioned. Oral disk transversely ellipsoid. Lower lip not reaching transverse line between gill openings. Lower lip covered with numerous small papillae. Maxillary barbel developed. Mouth relatively large. Premaxillary teeth 40−61 per ramus; dentary teeth 28−69 per ramus. Teeth bifid, medial cusp large and rounded, lateral cusp minute and pointed. Wide jaws, dentary bones forming an oblique angle, premaxillary bones almost co-linear.

Dorsal fin II,7, origin approximately at midpoint between pectoral- and pelvic-fin origins, last dorsal-fin ray reaching adipose fin when depressed. Pectoral fin I,6, spine tip curved inward, covered with enlarged odontodes distally; depressed tip reaching one-third length of pelvic-fin spine. Pelvic fin I,5, spine tip curved inward, almost reaching anal-fin origin when depressed. Anal fin I,5, spine tip straight, reaching seventh plate posterior to its origin. Caudal fin I,7−I,7, distal margin concave, inferior lobe longer than superior. Adipose fin with lightly curved spine, preceded by single median preadipose plate.

#### Color in life.

**Figure 3.**
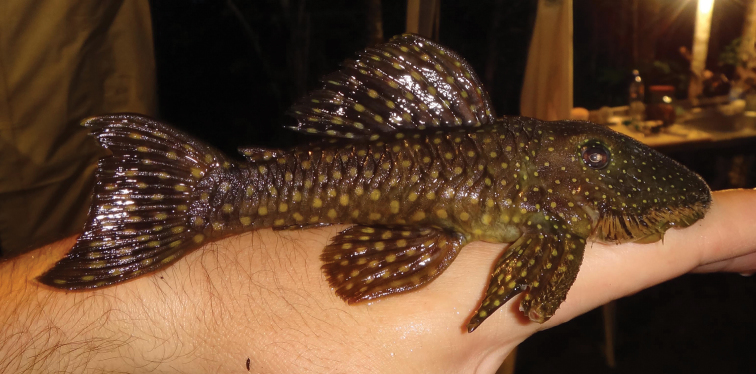
*Pseudancistrus zawadzkii*, live specimen, LBP 15045, paratype, female, 128.7 mm SL, Tapajós river, Pará State, Brazil.

Ground color dark greenish-brown on dorsum and sides of body, becoming dark brown posteriorly, and lighter brown ventrally. Anterior portion of head to posterior margin of orbits with many small, crowded, yellow spots; spots becoming abruptly larger on posterior portion of head, continuing on body, becoming slightly and gradually larger towards caudal peduncle. Dorsal plate series usually with two large spots per plate. Mid-dorsal plates usually with one large spot per plate. Lateral median plates with one large spot per plate. Mid-ventral plates and ventral plates with one large spot per plate. Dorsal-fin spine, rays and membranes with large round large spots. Adipose-fin with two large spots on spine and membrane. Pectoral, pelvic, anal and caudal fin with numerous and similarly sized yellow spots. Hypertrophied odontodes along head margin yellowish (Fig. 3).

#### Color in alcohol.

Similar to pattern described for living individuals, but with ground color dark brown, and spots pale tan (Fig. 1).

#### Sexual dimorphism.

Males possess a papilla posterior to urogenital opening, an attribute absent in females. Both sexes in *Pseudancistrus zawadzkii* exhibit highly hypertrophied odontodes along snout margin, similar to others species of *Pseudancistrus* (Armbruster 2004b). In some loricariid species of genus *Pareiorhaphis* those hypertrophied odontodes may be sexually dimorphic (Pereira et al. 2007), an attribute not observed in the new species *Pseudancistrus zawadzkii*.

#### Etymology.

Specific name is in honor of Cláudio Henrique Zawadzki, professor at Universidade Estadual de Maringá (UEM), Maringá, Paraná State, Brazil, in recognition of his dedication and remarkable contributions to the study of the family Loricariidae.

#### Distribution.

**Figure 4.**
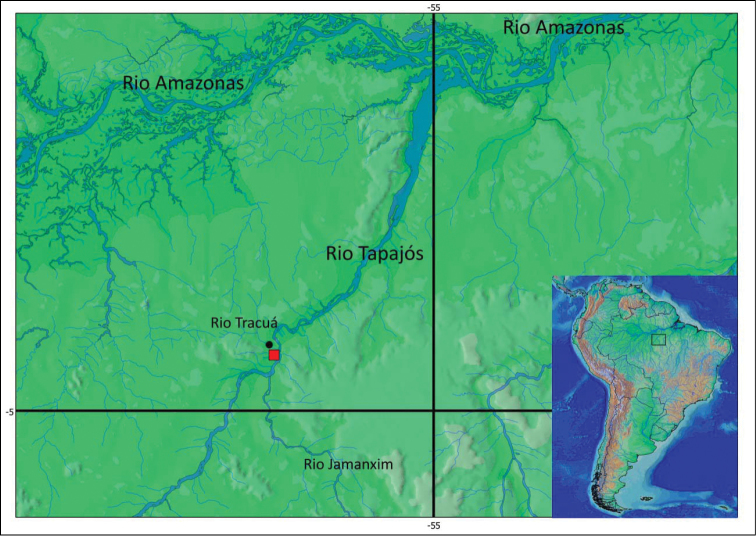
Map showing the type locality (red square) of *Pseudancistrus zawadzkii* at rio Tapajós, 04°33'09.7"S, 56°17'59.6"W, and paratype locality (black circle) at rio Tracuá, Tapajós river basin, 04°28'11.2"S, 56°17'01.1"W.

*Pseudancistrus zawadzkii* is known from rio Tapajós (04°33'10"S, 56°18'W) and rio Tracuá (04°28'11"S, 56°17'01"W), municipality of Itaituba, all from rio Tapajós basin, Pará State, Brazil. (see Fig. 4 for distribution map of type species localities).

#### Ecological notes.

**Figure 5.**
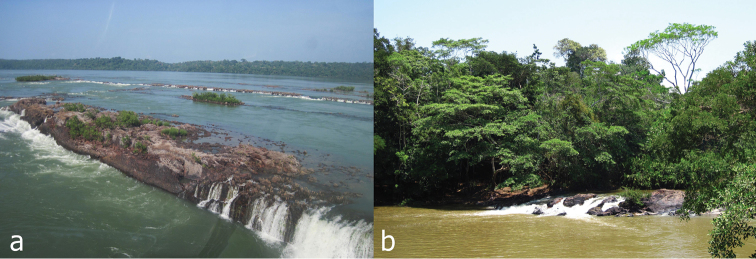
**a** Habitat at type locality of *Pseudancistrus zawadzkii*: rio Tapajós, municipality of Itaituba, Pará State, Brazil **b** habitat at paratype locality: rio Tracuá, Tapajós river basin, municipality of Itaituba, Pará State, Brazil.

The rio Tapajós, and rio Tracuá where *Pseudancistrus zawadzkii* occurs are clear water rivers, varying from medium to large size, with rocky outcrops forming small waterfalls and substrates of rocks and sand (Fig. 5).

### Phylogenetic analysis

Partial sequences of the nuclear gene F-reticulon 4 (RTN4) were obtained in this study and from GenBank for 44 specimens representing 35 Loricariidae species and the new species *Pseudancistrus zawadzkii* (Table 3). We included samples of the four lineages of *Pseudancistrus* proposed by Covain and Fisch-Muller (2012) to test whether *Pseudancistrus zawadzkii* is part of the *Pseudancistrus barbatus* group. *Corydoras oiapoquensis* Nijssen, 1972 (Callichthyidae) was used to root the phylogeny. Additionally, samples of Delturinae (*Hemipsilichthys gobio* Lutken, 1874) and Loricariinae (*Harttia guianensis* Rapp Py-Daniel & Oliveira, 2001) were included in the analysis as additional outgroups. The combined sequence data resulted in a matrix with 2,318 base pairs (bp), out of which 1,079 were conserved and 896 were variable. The estimated index of substitution saturation (Iss) performed in DAMBE 5.2.31 (Xia and Xie 2001) showed that the data was not saturated (i.e. Iss.c value greater than Iss).

Evolutionary relationships among species of *Pseudancistrus* sensu lato and other members of Otothyrini are similar between our ML phylogenetic tree (-lnL = 11470.59) and the one proposed by Covain and Fisch-Muller (2012). In our analysis, the genus *Pseudancistrus* is paraphyletic with species assigned to three different lineages. The first lineage is monotypic, composed of *Pseudancistrus genisetiger*, sister to *Hemipsilichthys gobio*, an outgroup taxon. Covain and Fisch-Muller (2012) suggested that *Pseudancistrus genisetiger* represents an undescribed genus within Delturinae. The second lineage of *Pseudancistrus* (*Pseudancistrus sidereus* + *Pseudancistrus pectegenitor*) is sister to a species of *Lithoxus* Eigenmann, 1910; Covain and Fisch-Muller (2012) suggested that the two species represent an undescribed genus or may be included in *Lithoxus*. The third lineage is composed of members of the *Pseudancistrus barbatus* group (*Pseudancistrus depressus*, *Pseudancistrus barbatus*, *Pseudancistrus corantijniensis*, *Pseudancistrus nigrescens*, the new species *Pseudancistrus zawadzkii* and an undescribed species from the rio Xingu known as L17 among hobbyists). The *Pseudancistrus barbatus* group forms a polytomy with almost all species analyzed in the ingroup (Fig. 3), and was recognized by Covain and Fisch-Muller (2012) as true *Pseudancistrus* since this group includes the type species *Pseudancistrus barbatus*. Additionally, Covain and Fisch-Muller (2012) revalidated two genera for several species previously assigned to *Pseudancistrus*, − *Lithoxancistrus* (for *Pseudancistrus orinoco* (Isbrücker, Nijssen & Cala, 1988)) and *Guyanancistrus* (for *Pseudancistrus* sp., *Pseudancistrus brevispinis* (Heitmans, Nijssen & Isbrücker, 1983), *Pseudancistrus longispinis* (Heitmans, Nijssen & Isbrücker, 1983) and *Pseudancistrus niger* (Norman 1926)). Our analysis also supports the recognition and composition of those two genera.

## Discussion

### Taxonomy and phylogenetic comparison

The new species *Pseudancistrus zawadzkii* possesses hypertrophied odontodes along the snout margin and lacks evertible cheek plates. Armbruster (2004b) identified that among Ancistrini, only *Pseudolithoxus*, *Lithoxancistrus*, and some members of *Guyanancistrus* and *Pseudancistrus* share the presence of hypertrophied odontodes along the snout in both sexes. Armbruster (2004b) also suggested that the species of *Pseudancistrus* that present this characteristic are derived; those species correspond to the *Pseudancistrus barbatus* group proposed by Chambrier and Montoya-Burgos (2008). Therefore, the new species described herein is a typical member of this group *sensu* Covain and Fisch-Muller (2012). Our phylogenetic analysis (Fig. 3) supports that hypothesis, and places the new species in a polytomy with *Pseudancistrus corantijniensis*, *Pseudancistrus* sp. L17 (undescribed species) and *Pseudancistrus nigrescens*, within the *Pseudancistrus barbatus* group. Our likelihood-based tests strongly rejected alternative topologies placing the new species in *Lithoxancistrus*, *Guyanancistrus* or with other species of *Pseudancistrus* apart from the *Pseudancistrus barbatus* group (see Table 2).

**Table 2.** Likelihood-based tests for alternative topologies. SH and AU are probability values obtained from the Shimodaira-Hasegawa and the Approximately Unbiased tests (Shimodaira 2002). Asterisks denote significant values (P<0.05 for SH and P<0.01 for AU and ELW) that imply the topology is rejected.

Test	Topology	- Ln *L*	∆ - Ln *L*	ELW	SH	AU
	ML	11910.81				
**1**	*Pseudancistrus zawadzkii* sister group to *Pseudancistrus pectegenitor* + *Pseudancistrus sidereus* ^a^	11952.41	41.60	<0.001*	0.021*	<0.001*
**2**	*Pseudancistrus zawadzkii* sister group to *Guyanancistrus* members ^a^	11962.24	51.43	<0.001*	0.011*	<0.001*
**3**	*Pseudancistrus zawadzkii* sister group to *Lithoxancistrus* members ^a^	11966.25	55.44	<0.001*	<0.001*	<0.001*
**4**	*Pseudancistrus zawadzkii* sister group to *Pseudancistrus genisetiger* ^a^	12033.30	122.49	<0.001*	<0.001*	<0.001*

^a^ The alternative topology was defined as the ML tree forcing the desired relationship.

**Table 3.** Taxa list, specimen and sequence data analyzed in the present study (n=44). Institutional acronyms follow Fricke and Eschmeyer (2013).

Species	Catalog Number	Field Number	GenBank Nº F-RTN4	Ref.
*Corydoras oiapoquensis*	MHNG 2682.023	GF06-186	GU210997	Alexandrou et al. (2011)
*Hemipsilichthys gobio*	LBP 2368	15363	EU817547	Chiachio et al. (2008)
*Harttia guianensis*	MHNG 2643.016	GF00–351	FJ013232	Chiachio et al. (2008)
*Hypostomus* sp.	MHNG 2721.062	PE08-198	JN855790	Covain and Fisch-Muller (2012)
*Hypostomus boulengeri* (Eigenmann & Kennedy, 1903)	MHNG 2519.23	ASU7	EU817560	Chiachio et al. (2008)
*Hypostomus gymnorhynchus* (Norman, 1926)	MHNG 2621.098	SU01-160	JN855789	Covain and Fisch-Muller (2012)
*Ancistrus cirrhosis* (Valenciennes, 1836)	MHNG 2645.037	MUS 202	HM623638	Rodriguez et al. (2011)
*Dekeyseria picta* (Kner, 1854)	MHNG 2588.046	MUS 162	JN855755	Covain and Fisch-Muller (2012)
*Dekeyseria scaphirhyncha* (Kner, 1854)	AUM 43874	V5528	JN855756	Covain and Fisch-Muller (2012)
*Hemiancistrus medians* (Kner, 1854)	MHNG 2664.078	GF00-084	JF747011	Fisch-Muller et al. (2012)
*Guyanancistrus brevispinis*	MHNG 2725.099	GF00-103	JN855772	Covain and Fisch-Muller (2012)
*Guyanancistrus brevispinis*	MHNG 2621.073	SU01-121	JN855773	Covain and Fisch-Muller (2012)
*Guyanancistrus longispinis*	MHNG 2725.100	GF99-204	JN855757	Covain and Fisch-Muller (2012)
*Guyanancistrus niger*	MHNG 2722.089	GF99-185	JN855759	Covain and Fisch-Muller (2012)
*Guyanancistrus* sp.	MHNG 2679.099	MUS 300	JN855774	Covain and Fisch-Muller (2012)
*Hopliancistrus tricornis* Isbrücker & Nijssen, 1989	MHNG 2588.051	MUS 146	JN855765	Covain and Fisch-Muller (2012)
*Lasiancistrus* aff. *caucanus*	MHNG 2586.043	MUS 118	JN855786	Covain and Fisch-Muller (2012)
*Lasiancistrus heteracanthus* (Günther, 1869)	MHNG 2613.037	CA 013	JN855787	Covain and Fisch-Muller (2012)
*Lasiancistrus planiceps* (Meek & Hildebrand, 1913)	STRI-01805	Stri 3526	JN855785	Covain and Fisch-Muller (2012)
*Lasiancistrus saetiger* Armbruster 2005	MHNG 2602.016	BR98-148	JN855754	Covain and Fisch-Muller (2012)
*Lasiancistrus schomburgkii* (Günther, 1869)	MHNG 2651.009	PE08-719	JN855782	Covain and Fisch-Muller (2012)
*Lasiancistrus schomburgkii*	MHNG 2651.068	GY04-308	JN855783	Covain and Fisch-Muller (2012)
*Lasiancistrus schomburgkii*	MHNG 2710.055	PE08-277	JN855784	Covain and Fisch-Muller (2012)
*Lasiancistrus tentaculatus* Armbruster, 2005	MhnG uncat.	MUS 573	JN855788	Covain and Fisch-Muller (2012)
*Lithoxus lithoides* Eigenmann, 1912	MHNG 2651.087	GY04-136	JN855777	Covain and Fisch-Muller (2012)
*Lasiancistrus pallidimaculatus* Boeseman, 1982	MHNG 2621.066	SU01-096	JN855778	Covain and Fisch-Muller (2012)
*Lasiancistrus planquettei* Boeseman, 1982	MHNG 2722.060	GF03-055	JN855779	Covain and Fisch-Muller (2012)
*Lithoxancistrus orinoco*	AUM 43725	V5246	JN855766	Covain and Fisch-Muller (2012)
*Lithoxancistrus orinoco*	AUM 42179	P4527	JN855767	Covain and Fisch-Muller (2012)
*Pseudancistrus barbatus*	MHNG 2653.059	GF00-074	JN855761	Covain and Fisch-Muller (2012)
*Pseudancistrus corantijniensis*	MHNG 2672.092	SU05-296	JN855781	Covain and Fisch-Muller (2012)
*Pseudancistrus depressus*	MHNG 2674.026	SU05-020	JN855780	Covain and Fisch-Muller (2012)
*Pseudancistrus genisetiger*	MHNG 2593.061	MUS 173	JN855764	Covain and Fisch-Muller (2012)
*Pseudancistrus nigrescens*	MHNG 2651.069	GY04-313	JN855770	Covain and Fisch-Muller (2012)
*Pseudancistrus nigrescens*	MHNG 2650.087	GY04-260	JN855771	Covain and Fisch-Muller (2012)
*Pseudancistrus pectegenitor*	AUM 42202	V5363	JN855769	Covain and Fisch-Muller (2012)
*Pseudancistrus pectegenitor*	ANSP 182801	V5433	JN855768	Covain and Fisch-Muller (2012)
*Pseudancistrus sidereus*	AUM 43443	P4871	JN855775	Covain and Fisch-Muller (2012)
*Pseudancistrus sidereus*	AUM 42180	P4537	JN855776	Covain and Fisch-Muller (2012)
*Pseudancistrus zawadzkii*	LBP 15045	61628	KJ028080	Present study
*Pseudancistrus* sp. L17	MHNG 2586.046	MuS 132	JN855763	Covain and Fisch-Muller (2012)
*Pseudolithoxus* cf. *kelsorum*	MHNG 2679.043	MUS 260	JN855762	Covain and Fisch-Muller (2012)
*Pseudancistrus dumus* (Armbruster & Provenzano, 2000)	MHNG 2708.080	MUS 288	JN855760	Covain and Fisch-Muller (2012)
*Pseudancistrus tigris* (Armbruster & Provenzano, 2000)	AUM 42215	V5292	JN855758	Covain and Fisch-Muller (2012)

*Pseudancistrus zawadzkii*, *Pseudancistrus corantijniensis*, and *Pseudancistrus nigrescens* share the presence of whitish colored snout odontodes and a dark colored body covered with white spots. The new species can be easily distinguished from *Pseudancistrus corantijniensis* and *Pseudancistrus nigrescens* by having large hypertrophied odontodes on the posteriormost portion of the non-evertible check plates, and marginal odontodes that increase gradually in length from tip of snout to cheeks. *Pseudancistrus barbatus* and *Pseudancistrus depressus* share reddish-brown snout odontodes, a probable synapomorphy, and are the sister group to *Pseudancistrus zawadzkii*, *Pseudancistrus corantijniensis* and *Pseudancistrus nigrescens*. Covain and Fisch-Muller (2012) suggested that *Pseudancistrus guentheri* and *Pseudancistrus kwinti* may be added to the *Pseudancistrus barbatus* group. However, those two species have a different body coloration pattern (Chambrier and Montoya-Burgos 2008; see fig. 3); in *Pseudancistrus kwinti* the body is either mottled or with bars, while in *Pseudancistrus guentheri* the body plates are dark at the base and pale along the edges (Willink et al. 2010).

### Biogeography and dispersal routes

**Figure 6.**
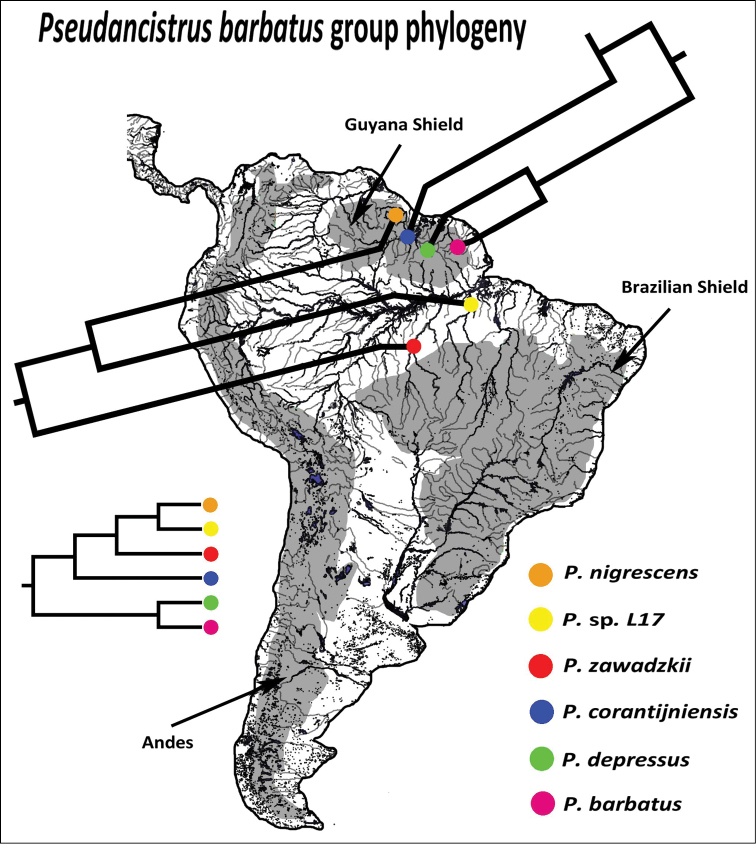
Distribution and phylogenetic relationships of species of the *Pseudancistrus barbatus* group based on F-reticulon 4 gene. Based in our first hypothesis of extand-species distribution of this group the ancestral was widespread through all Guyana Shield rivers and Amazon Brazilian Shield rivers, the species *Pseudancistrus zawadzkii* and *Pseudancistrus* sp. L17 are in the limited distribution of this group in Tapajós and Xingu rivers, drainages of Brazilian Shield into Amazon.

Named species of the *Pseudancistrus barbatus* group are distributed in rivers draining to Guyana Shield into the Atlantic Ocean, and the new species described herein is from Tapajós river draining of Brazilian Shield into the Amazon. In our phylogeny, species from the eastern Guyana Shield (*Pseudancistrus barbatus* and *Pseudancistrus depressus*) form a clade sister to a group composed of species from the western Guyana Shield (*Pseudancistrus corantijniensis* and *Pseudancistrus nigrescens*) and Amazon basin (*Pseudancistrus zawadzkii* and *Pseudancistrus* sp. L17) (Fig. 6). Therefore, based on this interpretation and our results of phylogenetic analysis, we suggested two hypotheses that could generate the distribution pattern of *Pseudancistrus barbatus* group extant-species. The first hypothesis is that the ancestral stock of the *Pseudancistrus barbatus* group was widely distributed through all Guyana Shield rivers and Amazon Brazilian Shield rivers, and the species *Pseudancistrus zawadzkii* and *Pseudancistrus* sp. L17 are in the limit of the distribution for the group in Tapajós and Xingu rivers, respectively. Gaston (1998) and Hubbell (2001) suggested that when allopatric divergence is the dominant mode of speciation, many daughter species are expected to arise from geographically widespread ancestral species. This is a reasonable interpretation given that named species of the group are widespread in rivers draining Guyana Shield into the Atlantic Ocean; the new species *Pseudancistrus zawadzkii* are from Tapajós river drainage of Amazon Brazilian Shield; the possible new and undescribed species *Pseudancistrus* sp. L17 are from Xingu river which also belongs to drainages of Amazon Brazilian Shield and others possible new and undescribed species of *Pseudancistrus barbatus* group may be present in drainages of Guyana Shield into Amazon (*Pseudancistrus* sp. L220 from rio Paru; *Pseudancistrus* sp. L251 from rio Cuminá (rio Erepecuru); *Pseudancistrus* sp. L383 from rio Trombetas; *Pseudancistrus* sp. L440 from rio Jatapu (Seidel 2008)). However, phylogenetic and taxonomic studies are necessary to confirm that the latter undescribed species belong to *Pseudancistrus barbatus* group.

**Figure 7.**
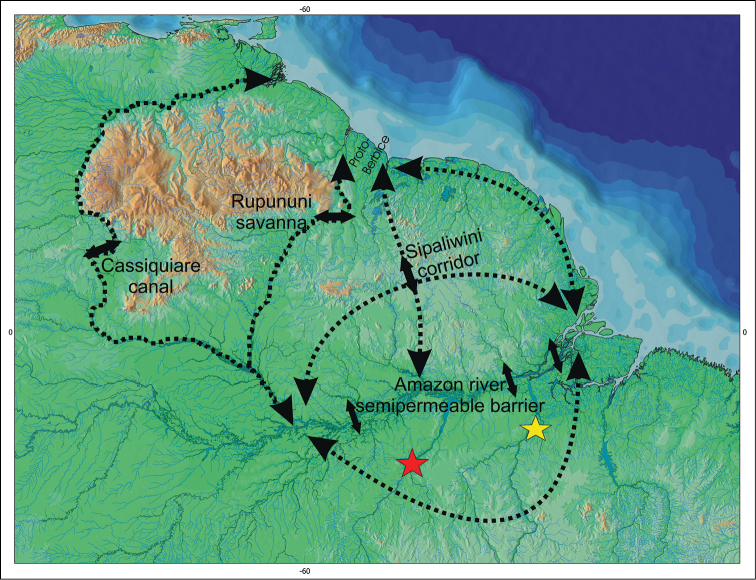
Hypothesized dispersal routs between basins of the Guiana Shield and Amazon Shield of ancestror of the *Pseudancistrus barbatus* group (based on Lujan and Armbruster 2011). Our second hypothesis of the *Pseudancistrus barbatus* group extent-species distribution is based on the assumption of a widespread ancestral through all Guyana Shield rivers and dispersal events enable the ancestor of *Pseudancistrus zawadzkii* (red star) and *Pseudancistrus* sp. L17 (yellow star) to colonize the Amazon Brazilian Shield rivers in Tapajós and Xingu rivers.

The second hypothesis suggests that the ancestral stock of *Pseudancistrus barbatus* group should have been distributed through Guyana Shield rivers and there existed several dispersal routes through Guyana and Amazon rivers, permitting that the ancestral lineages of *Pseudancistrus* sp. L17 and *Pseudancistrus zawadzkii* reached the rivers of Amazon basin (see Fig. 7 for dispersal routes). Therefore, examples of connections and areas of movement among Guyana drainages and the north tributaries of Amazon basin was reported by several authors: (1) the Rupununi portal, an example of seasonal connection among Takutu and Rupununi rivers (Armbruster and Werneke 2005; Lujan and Armbruster 2011; De Souza et al. 2012); (2) the corridor among Sipalawini (Corantijn river basin) and the Paru do Oeste (Amazon basin), also connected only in the rainy season (Nijssen 1972; Lujan and Armbruster 2011); (3) the Cassiquiare Canal, a large and permanently navigable corridor between the upper Orinoco and the upper Rio Negro (Amazon) (Chernoff et al. 1991; Buckup 1993; Schaefer and Provenzano 1993; Lovejoy and Araújo 2000; Turner et al. 2004; Moyer et al. 2005; Willis et al. 2007; Winemiller et al. 2008; Winemiller and Willis 2011); (4) Proto-Berbice, a river system which had its headwaters in an ancient mountain range draining northward to Guyana system (Rupununi and Essequibo rivers) and suffered a major sedimentation, erosion and/or corrosion of the highlands and at the end of the Pliocene had its head waters captured by the Amazon system; (5) the Atlantic coastal corridors resulted in a coastal marine corridor with reduced salinity due to the westerly Amazon River discharge, coastal junctions during times of marine regressions and expanded coastal plains, and stream captures (Eigenmann 1912; Boeseman 1968; Cardoso and Montoya-Burgos 2009; Lujan and Armbruster 2011).

Additionally, the mainstream of Amazon River can act as a permeable barrier for endemic taxa on the respective Guiana and Brazilian shields. Several genera known to tolerate more lowland conditions (e.g. *Ancistrus* Kner, 1854, *Lasiancistrus*, and *Hypostomus* Lacepéde, 1803) may be able to cross the Amazon basin, but such dispersal is unlikely among most species of Ancistrini (Lujan and Armbruster 2011). Also historically, epochs of cooler climate, as during glacial periods, could produce reduced precipitation, marine regressions, expansion of the coastal plain, and deepening of river channels. During such arid periods, rapids would have been more widespread, and deep-channel habitats that may currently work as barriers to fish dispersal would have been reduced (Schubert et al. 1986; Latrubesse and Franzinelli 2005; Lujan and Armbruster 2011). Drier climate will hardly change the Amazon river in a rapid, but can reduce its water flow allowing fish dispersal. Among Neotropical fishes *Psectrogaster essequibensis* Günther, 1864 (Characiformes: Curimatidae; see Vari (1987)), *Parotocinclus aripuanensis* Garavello, 1988, and *Pseudancistrus britskii* Boeseman, 1974 (Loricariidae: Hypoptopomatinae) are species known to support dispersal via the northern Brazilian Shield.

Also, the dispersal routes around adjacent drainages of southern and northern Guyana Shield and northern parts of the Brazilian Shield could allow the dispersal of the ancestral form of *Pseudancistrus zawadzkii* and *Pseudancistrus* sp. L17, as well as others ancestral species of the *Pseudancistrus barbatus* group and even species of Ancistrini (Lujan and Armbruster 2011). The movement of fish species around adjacent drainages could be explained by two hydrographic reconfiguration process: headwater capture events (geomorphological phenomenon) and marine regressions (sea level oscillation). Changes in the earth’s surface involving changes in the courses of rivers, as stream captures, portions of tributaries of a river in a watershed could be “captured” by adjacent basins resulting in isolated populations and at the same time letting species to move, or disperse, between adjacent drainages (Almeida and Carneiro 1998; Bishop 1995; Wilkinson et al. 2006, 2010; Roxo et al. 2012). Montoya-Burgos (2003) hypothesized that dispersal (followed by allopatric population divergence) among Amazon and North-eastern coastal rivers probably occurred by temporary connections between adjacent rivers during periods of lower sea level about 6–5 Ma (see fig. 5 in Montoya-Burgos 2003). Cardoso and Montoya-Burgos (2009) suggested the same process to explain dispersal of *Pseudancistrus brevispinis* along coastal rivers of the Guyana. Therefore, temporary lowland connections and headwater capture events, together with the previously related hypothesis of colonization routes, likely explain the widespread distribution of the *Pseudancistrus barbatus* group extant species on Guyana and Brazilian Shields, as well as how the ancestral lineages of *Pseudancistrus zawadzkii* and *Pseudancistrus* sp. L17 reached the drainages of the northern Brazilian Shield, in Tapajós and Xingu rivers.

### Comparative material

*Pseudancistrus barbatus* (Valencienes, 1840): ANSP 177366, 2, 76.5−103.7 mm SL, Burro Burro river, Water Dog Falls, Essequibo river basin, Guyana. ANSP 189119, 3, 75.1−151.5 mm SL, Lawa river, Sipalawini, Suriname. *Pseudancistrus brevispinis* (Heitmans, Nijssen & Isbrücker, 1983): ANSP 189128, 3, 56.8−125.7 mm SL, Marowini river, Sipalawini, Suriname. *Pseudancistrus nigrescens* Eigenmann, 1912: ANSP 177379, 5, 96.4−133.5 mm SL, Burro Burro river, Water Dog Falls, Essequibo river basin, Guyana. *Pseudancistrus orinoco* (Isbrücker, Nijssen & Cala, 1988): ANSP 160600, 6, 68.0−78.5 mm SL, Orinoco river, Venezuela. *Pseudancistrus pectegenitor* Lujan, Armbruster & Sabaj, 2007: ANSP 190755, 1, 206,2 mm SL, Ventuari river, Orinoco river basin, Venezuela. *Pseudancistrus sidereus* Armbruster, 2004b: ANSP 185321, 4, 148.6−154.1 mm SL, Casiquiari river, Venezuela. *Pseudancistrus* sp. L17: LBP 16551, 2, 75.3−101.0 mm SL; rio Xingu, Altamira, Pará State, Amazon river basin, Brazil. ANSP 193074, 3, 51.7−188.7 mm SL, Xingu river, Altamira, Pará State, Amazon river basin, Brazil. *Pseudancistrus* sp. ANSP 191153, 6, 49.2−75.7 mm SL, Ventuari river, Orinoco river basin, Venezuela.

## Supplementary Material

XML Treatment for Pseudancistrus zawadzkii
